# Supplementary material for: Transition to the Haldane phase driven by electron-electron correlations
Source: Nat Commun. 2023 Dec 22;14:8524. doi: 10.1038/s41467-023-44135-9 (PMC10740019; doi:10.1038/s41467-023-44135-9)
Supplement: Supplementary file 1 — Supplementary Info [file 41467_2023_44135_MOESM1_ESM.pdf]

SUPPLEMENTARY INFORMATION

Transition to the Haldane phase driven by electron-electron correlations

by A. Jażdżewska, M. Mierzejewski, M. Środa, A. Nocera, G. Alvarez, E. Dagotto, and J. Herbrych

SUPPLEMENTARY NOTE 1: GAP ANALYSIS.

In Supplementary Fig. 1, we present the finite-size $1/L$ and interaction U dependence of the $\Delta S = 2$ gap (the magnon gap $\Delta_{\rm S}/J$) for various values of the Hund exchange $J_{\rm H}/U = 0.05, 0.10, \dots, 0.40$. The main text displays the results of $1/L$ extrapolations of this data in Fig. 2B.

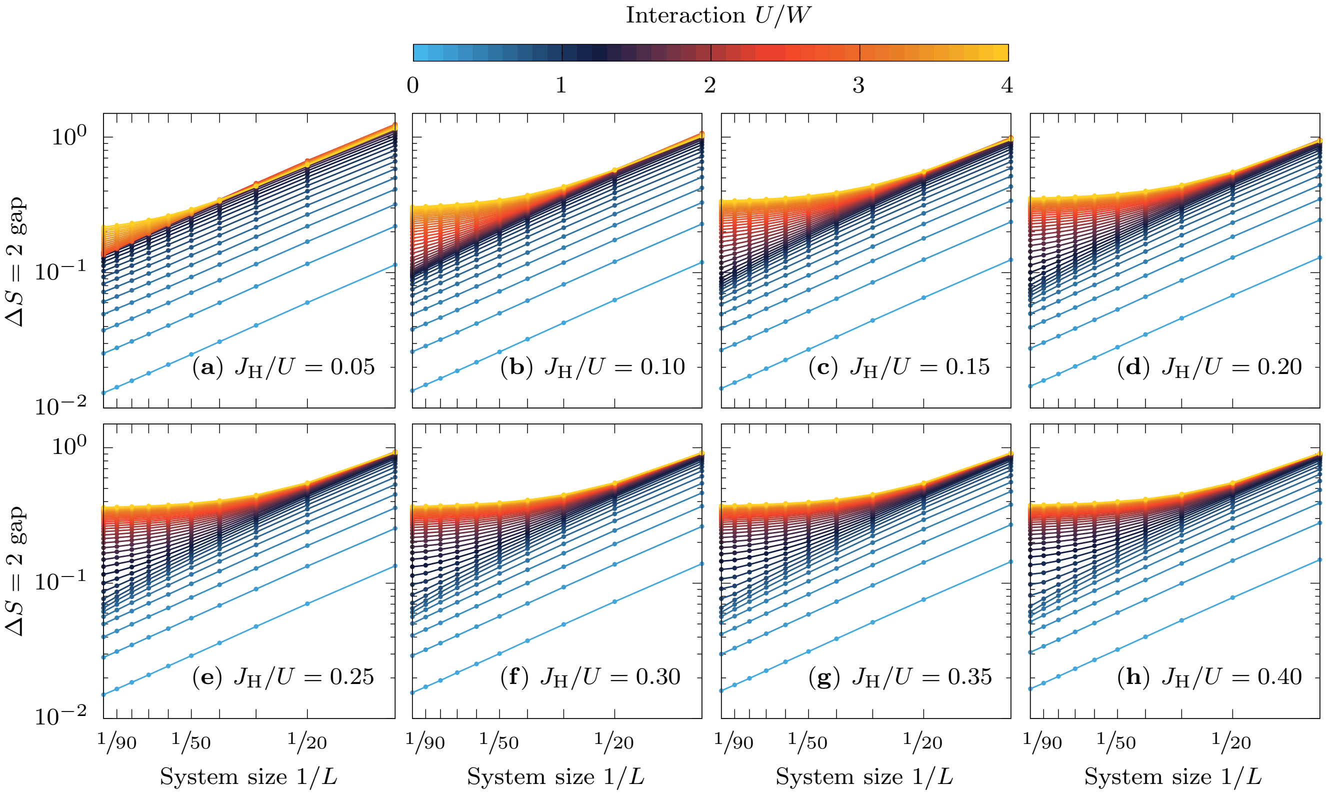

Supplementary Figure 1. **Spin gaps.** Finite-size scaling of $\Delta S = 2$ excitations (magnon gaps) for $J_{\rm H}/U = 0.05, 0.10, \dots, 0.40$ and $L \in \{10, 20, \dots, 100\}$. Line color-code represents the value of interaction U. All data in units of spin exchange $J = 2t^2/(U + J_{\rm H})$. The saturation to the finite value (to the Haldane gap $\Delta_{\rm S}/J \simeq 0.41$) is clearly visible in all panels.

SUPPLEMENTARY NOTE 2: STATIC AND DYNAMIC SPIN CORRELATIONS.

In the main text, we have described how the dynamical spin-spin correlation, i.e., the non-local Green's function $\langle\langle T_1^z T_\ell^z \rangle\rangle_{\omega=0}$, behave vs. distance ℓ and strength of the interaction U. Here, we present additional results for the static spin correlations, $\langle T_1^z T_\ell^z \rangle = \langle {\rm gs}| T_1^z T_\ell^z |{\rm gs}\rangle$.

In Supplementary Fig. 2, we present the analysis of the static $\langle T_1^z T_\ell^z \rangle$, similar to the one presented in Fig. 3 of the main text. As evident from panels (a-d), the overall behaviour of $\langle T_1^z T_\ell^z \rangle$ is almost identical to the zero-frequency $\omega = 0$ data. The main difference between the static and the dynamic correlation function can be observed in the extracted edge correlation length $\xi_{\rm e}$ close to the transition $U \sim U_{\rm c}$ [compare Fig. 3C of the main text and Supplementary Fig. 2(d)]. I.e., the edge correlation length extracted from $\langle\langle T_1^z T_\ell^z \rangle\rangle_{\omega=0}$ data is much sharper than the

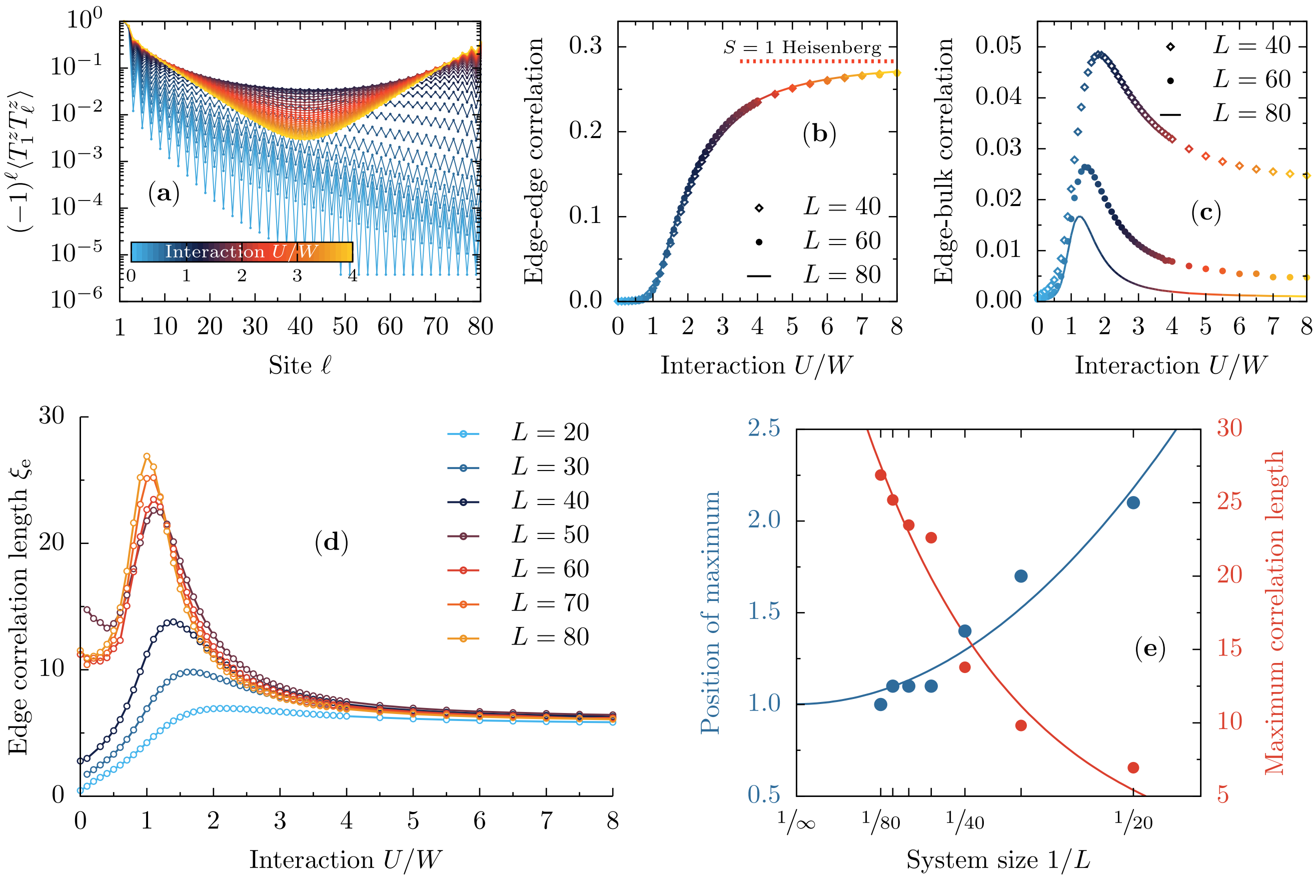

Supplementary Figure 2. **Static spin correlations.** (**a**) Distance ℓ dependence of static spin-spin correlations $(-1)^\ell \langle T_1^z T_\ell^z \rangle$ for various values of interaction U (denoted by color code). The results are normalized by the $\ell = 1$ value of the correlation function. (**b**) Edge-edge $|\langle T_1^z T_L^z \rangle|$ and (**c**) edge-bulk $|\langle T_1^z T_{L/2}^z \rangle|$ spin correlations vs. interaction strength U/W. At U_c, one observes the appearance of finite edge-edge correlations, saturating at $U \gg W$ to the value given by the $\mathcal{S} = 1$ Heisenberg model (red dashed line). (**d**) Edge correlation length ξ_e vs. interaction U strength, extracted from $(-1)^\ell \langle T_1^z T_\ell^z \rangle = a \exp(-\ell/\xi_e)$ for $\ell < L/2$. (**e**) System-size dependence of the position of (blue points, left y-axis) and the value (red points, right y-axis) of the maximum edge correlation length. All data calculated for $J_H/U = 0.25$.

one extracted from the static $\langle T_1^z T_\ell^z \rangle$. Nevertheless, the scaling with the system size [see Supplementary Fig. 2(e)] of the position of the maximum of ξ_e as well as the value itself indicate a transition at U_c.

To understand the difference between static and dynamic results, consider the sum rule relating (at zero temperature) both of these quantities, i.e.,

$$\langle T_m^z T_n^z \rangle = \int_0^\infty d\omega \, \langle\langle T_m^z T_n^z \rangle\rangle_\omega \, . \qquad (1)$$

It is evident that both approaches would yield the same behaviour if $\langle\langle T_m^z T_n^z \rangle\rangle_\omega = \delta(\omega)$. However, our analysis presented in Supplementary Fig. 3 indicates that the behaviour of the non-local Green's function $\langle\langle T_m^z T_n^z \rangle\rangle_\omega$ strongly depends on the pair of sites (m, n) considered. Note that, in the presented analysis, we have used finite broadening η [see Eq. (5)]. Consequently, all sharp features of the spectrum are broadened by a Lorentzian.

Within the fermionic Haldane phase, $U > U_c$, the spectrum of the bulk of the system, $m \sim n \sim L/2$, has only the incoherent part $\omega > 0$, and it is gapped; see Supplementary Fig. 3(a,b). Such behaviour is expected because (m, n) elements contribute (via the Fourier transform) to the overall dispersion relation presented in Fig. 1 of the main text. On the other hand, the correlation between the edge $m = 1$ and the rest of the system needs more attention: (i) consistent with the phenomenology of zero-energy edge modes, the edge-edge $(m, n) = (1, L)$ dynamical correlations contain only the $\omega = 0$ δ-function (broaden by the Lorentzian in our numerical investigation), see

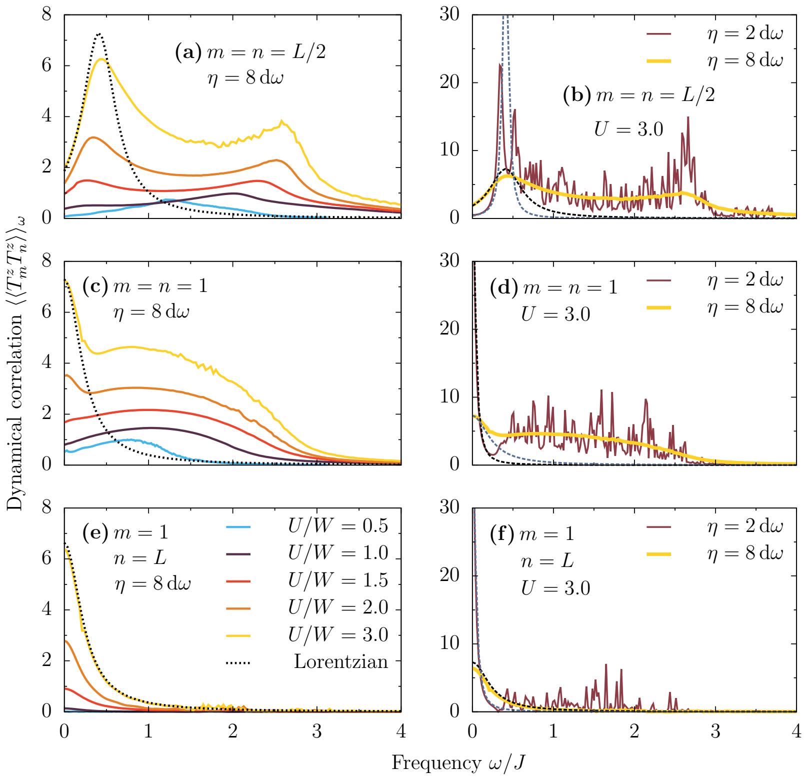

Supplementary Figure 3. **Dynamical spin correlations** $\langle\langle T^z_m T^z_n \rangle\rangle_\omega$**.** Shown are the frequency ω dependence of the local Green's function in (**a,b**) the bulk of the system $m = n = L/2$ and in (**b,c**) at the edge $m = n = 1$. (**d,e**) The non-local Green function between edges of the system ($m = 1, n = L$). In all plots, color solid curves represent data for various values of interaction $U/W = 0.5, 1.0, \ldots, 3.0$, $J_{\mathrm{H}}/U = 0.25$, and $\eta = 8\delta\omega$ (left column) and $\eta = 2\delta\omega$ (right column). The dashed line represents the Lorentzian.

Supplementary Fig. 3(c,d). Consequently $\langle T^z_1 T^z_\ell \rangle \simeq \langle\langle T^z_1 T^z_\ell \rangle\rangle_{\omega=0}$. (ii) Edge-bulk correlation $(1, \sim L/2)$ vanishes, again, consistent with the presence of the edge states (not shown). (iii) However, in the proximity of the edges (for $m \sim n \sim 1$), the dynamical correlations contain both coherent $\omega \sim 0$ (edge mode) and incoherent $\omega > 0$ parts [see Supplementary Fig. 3(e,f)], and $\langle T^z_1 T^z_{m\sim 1} \rangle \neq \langle\langle T^z_1 T^z_{m\sim 1} \rangle\rangle_{\omega=0}$.

Finally, in Supplementary Fig. 4, we present additional evidence for the topological phase transition at $U = U_{\mathrm{c}} \simeq 0.9W$ (for $J_{\mathrm{H}}/U = 0.25$) from spin correlation data.
(1) In panel (a), we compare the local Green's function $n = m = 1$ and non-local edge-edge correlations $n = 1\,, m = L$ at zero-frequency $\omega = 0$. The change in the local value is related to the development of the magnetic moment $\mathcal{S}$. On the other hand, the non-local (edge-edge) probes only the appearance of edge zero-modes (as discussed in the main text). It is evident from the presented results that both quantities merge at $U = U_{\mathrm{c}}$, consistent with the presence of the zero frequency edge modes for $U > U_{\mathrm{c}}$.
(2) In panels (b) and (c), we present the same data as in Fig. 3B of the main text and Supplementary Fig. 2(b), respectively, in a log-y scale. Evidently, for $U > U_{\mathrm{c}}$, the non-zero correlation functions do not originate from finite-size effects. On the other hand, for $U < U_{\mathrm{c}}$, the values of edge-edge correlations (both static and dynamic) decay exponentially with system size.

SUPPLEMENTARY NOTE 3: ENTANGLEMENT SPECTRUM.

Supplementary Fig. 5 depicts additional results for the entanglement spectrum $-2\ln\lambda_\alpha$. Consistent with the discussion presented in the main text, the value of the interaction U for which the spectrum is evenly degenerate moves to U_{c} with increasing system size L (see also Fig. 4 of the main text). Furthermore, it is evident from the presented results that the λ_α spectrum does not contain any apparent structure for $U < U_{\mathrm{c}}$, and the condition [i.e., $\ln\lambda_{i+1} - \ln\lambda_i \leq 0.01$] of evenly degenerate entanglement spectrum is not fulfilled.

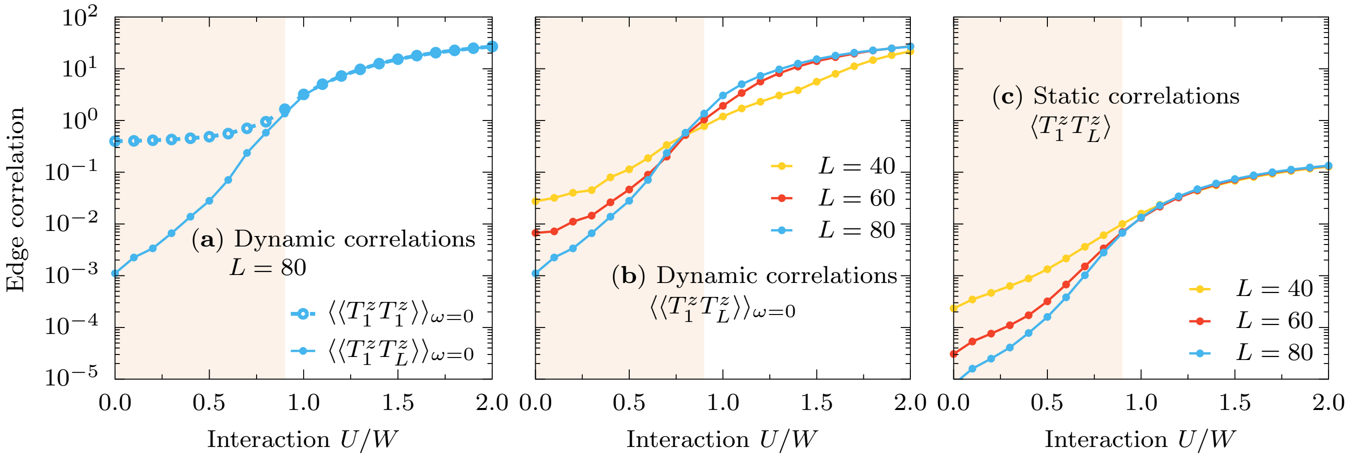

Supplementary Figure 4. **Spin correlations.** (**a**) Interaction U dependence of local $m = n = 1$ and non-local edge-edge $m = 1\,, n = L$ Green's functions $|\langle\langle T_1^z T_m^z \rangle\rangle_{\omega=0}|$ for $L = 80$ sites. (**b,c**) Interaction dependence of (**b**) dynamic and (**c**) static edge-edge spin correlations for various system sizes $L = 40, 60, 80$. In all panels, the shaded region represents the topologically trivial phase.

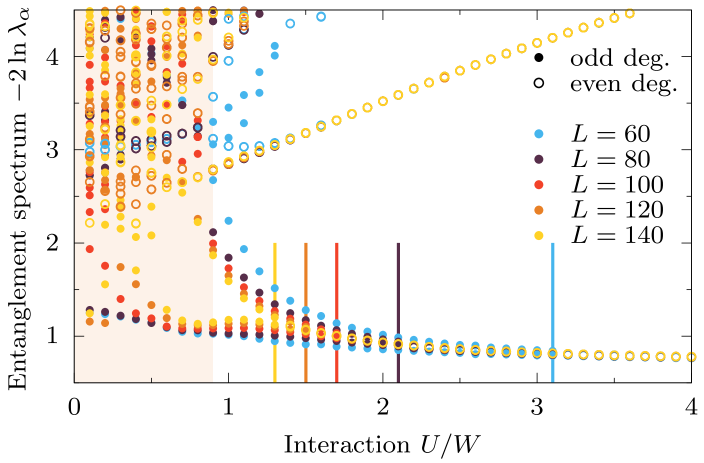

Supplementary Figure 5. **Entanglement entropy spectrum.** Interaction U/W dependence of the entanglement spectrum $-2\ln\lambda_\alpha$, obtained for $J_\mathrm{H}/U = 0.25$ and $L = 60, 80, \ldots, 140$ site systems divided in half. The vertical lines depict the value of interaction at which the lowest 50 eigenvalues λ_α are evenly degenerate. The shaded region depicts the topologically trivial phase.
